# Respiratory Variability during NAVA Ventilation in Children: Authors’ Reply

**DOI:** 10.3389/fped.2015.00013

**Published:** 2015-02-19

**Authors:** Hau-Tieng Wu, Florent Baudin, Martin G. Frasch, Guillaume Emeriaud

**Affiliations:** ^1^Department of Mathematics, University of Toronto, Toronto, ON, Canada; ^2^Department of Pediatrics, CHU Sainte-Justine, Université de Montréal, Montreal, QC, Canada; ^3^Department of Obstetrics and Gynecology, CHU Sainte-Justine Research Center, Université de Montréal, Montreal, QC, Canada; ^4^Department of Neurosciences, CHU Sainte-Justine Research Center, Université de Montréal, Montreal, QC, Canada; ^5^Centre de Recherche en Reproduction Animale, Université de Montréal, St-Hyacinthe, QC, Canada

**Keywords:** pediatric intensive care, mechanical ventilation, neurally adjusted ventilatory assist, diaphragm, children

We thank Dr. Mhanna for his interesting comment (1) on our recent article about respiratory variability during different pediatric ventilatory conditions (2). In this article, we described different patterns of respiratory drive variability depending on the ventilatory modes, using synchrosqueezing transform (SST) to gage changes in the variability of the electrical diaphragm activity (EAdi). In particular, neurally adjusted ventilatory assist (NAVA) was associated with a respiratory pattern most resembling that of the pattern observed in a separate group of “normally breathing” children, without ventilatory support or respiratory distress. Dr. Mhanna appropriately underlines the difficulty to draw definitive conclusions based on our study design, in particular, because of the *post hoc* nature of the analysis and the slight differences between the mechanically ventilated group and the control group. Indeed, the patients in the control group were smaller [median 3.9 kg (interquartile 3.5–5.0) vs. 5.7 kg (4.8–6.7), *p*  = 0.04] and tended to be younger [1.5 months (1–3) vs. 4.5 (2.5–4.7), *n.s*.] (2). These limitations were acknowledged and discussed in our article, and we certainly agree to consider this work as a hypothesis-generating study rather than as a definitive study.

Future studies will be needed to further address the impact of NAVA on the respiratory variability and it is important to discuss some possible future directions and/or pitfalls. Future works should be conducted prospectively and should assess the impact of ventilatory modes among comparable groups. Regarding variability analysis strategy, although the mathematical and statistical properties of SST have been well studied (3–5) and seem pertinent to analyze the respiratory signals and in particular EAdi, there is room to improve the current approach. First of all, the underlying mathematical model, adaptive harmonic model (3, 5), is limited to oscillatory signals with slowly varying instantaneous frequency and amplitude modulation. As an illustrative example, the cardiac R to R interval (RRI) between consecutive beats is seemingly irregular in normal subjects, which is often called “heart rate variability.” In other words, the frequency of heart beats is time-varying. However, in the extreme case like atrial fibrillation, the RRI behaves like noise – the heart rate changes fast from one beat to another. The time-varying frequency of a normal subject’s heart beats can be understood as slowly varying, while the one of a subject with atrial fibrillation can be understood as fast varying. In general, when the instantaneous frequency varies fast, the features captured from the signal by SST might not be predicted by the current theorem. The respiratory signals are oscillatory in nature, but since non-stationary changes could also occur (e.g., sudden apneas, crying, hiccup, etc), extending the algorithm to capture the fast varying instantaneous frequency is an important topic in the methodology development. Second, the intrinsic meaning of the NRR index is a quantification of how well the signal can be captured by the adaptive harmonic model. As its potential usefulness has been shown in several applications (6, 7), we should not forget that it just reflects a single facet of a complicated system. Other combinations of features extracted from the signal via SST and other techniques should be established in order to capture other aspects of our physiological system. One possible feature combination has been shown in the ventilator weaning prediction problem (8): the amplitude modulation and instantaneous frequency are combined to predict the weaning success rate.

Combining information from different signals would also be important. In our recent study, the two signals – pressure and EAdi – were analyzed separately. Only at the level of the statistical analysis was the pressure signal considered a covariate with the EAdi as dependent variable. A combination of information from these two channels, as early as at the level of mathematical bivariate signal analysis, could provide a finer dynamical feature inside physiology. However, finding a suitable technique to integrate information from multiple time series with complicated dynamics, both deterministic and stochastic, in particular in the medical field, is still a challenging problem. One possible approach to this goal is the currently proposed non-linear independent component analysis or empirical intrinsic geometry analysis (EIG) (9, 10), which has been successfully applied to a sleep dynamical study (11). Another important direction is taking other physiological signals into account. For example, the electrocardiographic signal (ECG) is ubiquitous in ICU, and certain neurocardiac features of physiological dynamics could be inferred from the heart rate variability.

Besides its major positive impact on patient ventilator synchrony (12–14) and its interest for promoting the patient ventilatory drive during mechanical ventilation (15), our preliminary data suggest that NAVA may also favor a “physiological” respiratory variability pattern (2). The proposed directions for future validation studies may permit to deeper appreciate and enhance the clinical potential of these respiratory variability modifications, in particular regarding the lung recruitment (16, 17).

## Conflict of Interest Statement

The authors declare that the research was conducted in the absence of any commercial or financial relationships that could be construed as a potential conflict of interest.

## References

[1] Mhanna MJ Impact of ventilatory modes on the breathing variability in mechanically ventilated infants: a commentary. Front Pediatr (2015) 2:147 10.3389/fped.2014.00147 25642420 PMC4294133

[2] Baudin F Wu HT Bordessoule A Beck J Jouvet P Frasch MG Impact of ventilatory modes on the breathing variability in mechanically ventilated infants. Front Pediatr (2014) 2:132. 10.3389/fped.2014.00132 25505779 PMC4242927

[3] Chen YC Cheng MY Wu HT Nonparametric and adaptive modeling of dynamic periodicity and trend with heteroscedastic and dependent errors. J R Stat Soc B (2014) 76(3):651–82 10.1111/rssb.12039

[4] Wu HT Instantaneous frequency and wave shape function (I). Appl Comput Harmonic Analysis (2013) 35:181–99 10.1016/j.acha.2012.08.008

[5] Daubechies I Lu J Wu HT Synchrosqueezed wavelet transforms: an empirical mode decomposition-like tool. Appl Comput Harmonic Analysis (2011) 30(1):243–61 10.1016/j.acha.2010.08.002

[6] Lin YT Wu HT Tsao J Yien HW Hseu SS. Time-varying spectral analysis revealing differential effects of sevoflurane anaesthesia: non-rhythmic-to-rhythmic ratio. Acta Anaesthesiol Scand (2014) 58(2):157–67. 10.1111/aas.12251 24410106

[7] Lin YT Hseu SS Yien HW Tsao J Analyzing autonomic activity in electrocardiography about general anesthesia by spectrogram with multitaper time-frequency reassignment. Proc IEEE-BMEI (2011) 2:628–32 10.1109/BMEI.2011.6098432

[8] Wu HT Hseu SS Bien MY Kou YR Daubechies I. Evaluating physiological dynamics via synchrosqueezing: prediction of ventilator weaning. IEEE Trans Biomed Eng (2014) 61(3):736–44. 10.1109/TBME.2013.2288497 24235294 PMC7309332

[9] Singer A Coifman RR Non-linear independent component analysis with diffusion maps. Appl Comput Harmonic Analysis (2008) 25(2):226–39 10.1016/j.acha.2007.11.001

[10] Talmon R Cohen I Gannot S Coifman RR Diffusion maps for signal processing: a deeper look at manifold-learning techniques based on kernels and graphs. IEEE Trans Signal Process (2013) 30(4):75–86 10.1109/MSP.2013.2250353

[11] Wu HT Talmon R Lo YL. Assess sleep stage by modern signal processing techniques. IEEE Trans Biomed Eng (2014). 10.1109/TBME.2014.2375292 25438301

[12] Baudin F Pouyau R Cour-Andlauer F Berthiller J Robert D Javouhey E. Neurally adjusted ventilator assist (NAVA) reduces asynchrony during non-invasive ventilation for severe bronchiolitis. Pediatr Pulmonol (2014). 10.1002/ppul.23139 25488197

[13] Bordessoule A Emeriaud G Morneau S Jouvet P Beck J. Neurally adjusted ventilatory assist improves patient-ventilator interaction in infants as compared with conventional ventilation. Pediatr Res (2012) 72(2):194–202. 10.1038/pr.2012.64 22580718

[14] Ducharme-Crevier L Beck J Jouvet P Neurally adjusted ventilatory assist (NAVA) allows patient-ventilator synchrony during pediatric non-invasive ventilation. A crossover physiological study. Crit Care (2015). 10.1186/s13054-015-0770-7 PMC4342194 25886793

[15] Kallio M Peltoniemi O Anttila E Pokka T Kontiokari T. Neurally adjusted ventilatory assist (NAVA) in pediatric intensive care-a randomized controlled trial. Pediatr Pulmonol (2015) 50(1):55–62. 10.1002/ppul.22995 24482284

[16] Blankman P Hasan D van Mourik MS Gommers D. Ventilation distribution measured with EIT at varying levels of pressure support and neurally adjusted ventilatory assist in patients with ALI. Intensive Care Med (2013) 39(6):1057–62. 10.1007/s00134-013-2898-8 23553568

[17] Suki B Alencar AM Sujeer MK Lutchen KR Collins JJ Andrade JS Jr Life-support system benefits from noise. Nature (1998) 393(6681):127–8 10.1038/30127 9603516

